# Ethanolic extract of *Mucuna pruriens* leaves ameliorates carbon tetrachloride and rifampicin-induced hepatotoxicity and nephrotoxicity in wistar albino rat

**DOI:** 10.1186/s12906-021-03455-3

**Published:** 2021-11-17

**Authors:** Temidayo Ogunmoyole, Ayomide Micheal Ola-Awe, Omotola Grace Fatile

**Affiliations:** 1 Department of Medical Biochemistry, College of Medicine, Ekiti State University, P.M.B., 5363, Ado Ekiti, Ekiti State Nigeria; 2 Department of Science Laboratory Technology, Faculty of Science, Ekiti State University, Ado Ekiti, Nigeria

**Keywords:** *Mucuna pruriens*, Carbon tetrachloride, Rifampicin, Oxidative stress

## Abstract

**Background:**

*Mucuna pruriens* (L.) has been used for the treatment of several ailments in folkloric medicine. The present study therefore investigates the hepatoprotective and nephroprotective potentials of its leaves extract with a view to providing a potent alternative in the management of liver and kidney diseases.

**Methodology:**

Forty male albino rats were randomly placed into eight groups comprising five animals each. Animals in group I were administered with the distilled water, while groups II and VI were exposed to CCl_4_ and rifampicin respectively. Animals in groups III and IV were initially exposed CCl_4_ and treated with 50 and 100 mg/kg bw *M. pruriens* respectively. Similarly, groups VII and VIII animals were exposed to rifampicin and treated with 50 and 100 mg/kg bw *M. pruriens* respectively. Animals in group V were treated with 100 mg/kg bw silymarin by oral gavage after an initial exposure to CCl_4_. Selected biomarkers of liver and kidney damage were determined in the serum and organs homogenate. Liver and kidney slices of experimental animals were also stained for histopathological examination.

**Results:**

Exposure to CCl_4_ and rifampicin respectively resulted in marked distortion in lipid profile, inhibition of antioxidant enzymes and a surge in ALT, AST, ALP, urea, uric acid, bilirubin and creatine kinase. Treatment with *M. pruriens* extract reversed all deranged biochemical and histopathological parameters in a dose-dependent manner.

**Conclusion:**

Extract of *M. pruriens* leaves restored deranged biochemical and histopathological parameters in the liver and kidney with similar potency to silymarin. Hence, leaf extract of *M. pruriens* is a potential hepatoprotective and nephroprotective agent that can be exploited in the management of liver and kidney diseases.

## Introduction

In recent times, oxidative stress concept has attracted vast research attention [1]. Primarily, oxidative stress results from an alteration in the fragile equilibrium between free radicals and antioxidants in the physiological system. Free radicals are highly reactive chemical species capable of causing pathological damage to critical macromolecules such as proteins, nucleic acids, lipids and carbohydrates [1, 2]. The etiology of almost all known diseases has been linked to free-radical induced oxidative stress. Hence, research interest has been tailored towards the identification, isolation and exploitation of agents (natural or synthetic) that can mitigate the deleterious effect of free radicals [3]. Carbon tetrachloride and rifampicin have been employed to create animal model of hepatotoxicity and nephrotoxicity respectively [4]. Toxicity of carbon tetrachloride (CCl_4_) involves serial bioactivation involving chloromethylation, saturation and peroxidation to trichloromethyl free radical (CCl_3_). Consequently, the trichloromethyl radical triggers a progressive deterioration of membrane phospholipid of the hepatic endoplasmic reticulum which results in structural and functional disruption of hepatocytes [4]. On the other hand, rifampicin exhibits its toxicity by activating pregnane X receptor (PXR), leading to an increased generation of reactive intermediates with hepatotoxic and nephrotoxic potential [5–7]. There are pieces of evidence suggesting that rifampicin acts by causing derangement in heme biosynthesis leading to accumulation of hepatotoxic protoporphyrin [7].

Medicinal plants have been recognized since antiquity as a veritable tool in the management of ailments [8]. Traditional herbal medicine has been embraced as an alternative to orthodox medicine in several developing and developed nations. This is partly due to the fact that herbs are natural and present with minimal side effect when administered [9]. Reports indicate that over 50% of drugs currently available were synthesized from proven medicinal plants [9, 10]. Globally, phytotherapy awareness and acceptance is growing steadily as it has been described as a return to nature in the treatment of diseases [11, 12].

*Mucuna pruriens* (L.) is a tropical plant with notable usage as herbal remedy for several ailments in many nations of the world [13]. Its seed extracts exhibit potent neuroprotective activity due to the presence of L-DOPA in significant amount. This explains its popular use in the management of Parkinson disease [14–17]. Seeds extract of *M. pruriens* also possess antihyperglycemic, antihyperlipidemic and antitumor potentials in animal models [18–20]. Reports have suggested that *M. pruriens* extract inhibits lipid peroxidation and enhances learning skills and memory [21–23]. Its anti-inflammatory, analgesic and antipyretic properties has been well documented [24–26]. Extract of *M. pruriens* seeds has been used in the management of rheumatoid arthritis, diabetes, atherosclerosis and sexual dysfunctions [27, 28]. *M. pruriens* seed extract has potent immunological properties which is perhaps the basis for its use as anti-venom [29].

Considering the enormous medicinal potentials of *M. pruriens* seeds*,* there is a dire need to investigate the therapeutic potential of its leaves extract in the management of liver and kidney diseases. This research effort might be a lead step in the provision of a potent alternative to conventional drugs currently in use for the treatment of these diseases.

## Materials and methods

### Plant materials

Fresh *Mucuna pruriens* (L.) leaves were obtained within the University campus, botanically identified at the Department of Plant Science, Ekiti State University, Ado-Ekiti. The voucher specimen of the leaf with number UHAE2020070 was deposited at the University herbarium. The air-dried leaves were then powdered using a Warring blender. Powdered leaf was weighed and stored in an airtight container.

### Reagents and chemicals

All reagents and chemicals were of high analytical grade obtained from standard commercial suppliers. All biochemical parameters were determined using Randox kit.

### Extraction of the extract

*Mucuna pruriens* (L.) leaves were air-dried for thirty (30) days at room temperature. The air-dried samples were ground to fine powder using a blender. 32.5 g of the powdered leaves was soaked in 100 ml of 80% ethanol and left for 72 h. It was then filtered using a cheese cloth to obtain a clear filtrate which was tightly covered with an insect-proof net and allowed for evaporation to dryness, which was monitored by weighing until a constant weight was obtained. The crude extract was kept airtight in a glass petri-dish inside a refrigerator and subsequently reconstituted with distilled water for animal treatment.

### Animals protocol

Ethical approval certificate (ORD/AD/EAC/19/0082) for the study was obtained from the Committee for Care and Use of Experimental Animals, Office of Research and Development, Ekiti State University, Ado Ekiti, Nigeria. Forty wistar albino rats weighing 150 g – 170 g were obtained from the animal house of the Department of Science Technology, The Federal Polytechnic, Ado-Ekiti and housed in clean wire meshed cages under standard conditions temperature (24 ± 1 °C), relative humidity (40–60%), and 12 / 12-h light and dark cycle. They were allowed to have free access to food (commercial palletized diet from Vital Feed Mill) and drinking water ad libitum daily. The rat beddings were changed and replaced every day throughout the experimental period. Carbon tetrachloride (3 ml/kg bw) was administered intraperitoneally to induce liver damage while rifampin (250 mg/kg bw) was administered by oral gavage. The experimental design is as indicated below:

#### Preparation of organs homogenate

After fourteen days of treatment, animals were fasted for 24 h prior to decapitated under very mild ether anesthesia and quickly dissected to obtain the liver and kidney. They were trimmed of fatty tissue, washed in saline, blotted with filter paper and weighed. Tissues were then chopped into bits and homogenized in ten volumes of the homogenizing phosphate buffer (pH 7.4) using a Teflon homogenizer. The resulting homogenates were centrifuged at 3000 rpm at 4 ^ο^C for 30 min. The supernatant obtained was collected and stored under 4 ^ο^C and then used for biochemical analyses.

#### Preparation of serum

Whole blood was collected into plain bottles by cardiac puncture and allowed to stand for 1 h to allow for coagulation. The coagulated blood was then centrifuged at 3000 rpm for 15 min at 25 ^ο^C to obtain a clear supernatant which was carefully decanted and placed on ice for the estimation of serum biochemical parameters.

### Assay for Creatine kinase (Ck-Mb) activity

Creatine kinase was assayed following the method previously described by Mattenheimer [30]. Briefly, 1.0 mL of imidazole buffer (10 mM, pH 6.6) containing: creatine phosphate (30 mM), glucose (20 mM), N-acetyl-cysteine (20 mM), magnesium acetate (10 mM), ethylene diaminetetraacetic acid (2 mM), ADP (2 mM), NADP (2 mM), AMP (5 mM), DAPP (10 μM), G6PDH (≥2.0 ku/L) and HK (≥2.15 ku/L) was pipetted into a thermostated cuvette and incubated at 37 ^ο^C. Thereafter, 50 μL of serum was added and thoroughly mixed. Absorbance of the resulting mixture at 340 nm was monitored for 5 min at 30 s interval.

### Assay of aspartate aminotransferase (AST) activity

Aspartate aminotransferase activity was determined as earlier described by Reitman and Frankel [31]. Briefly, 0.1 mL each of serum and organ homogenates respectively was mixed with phosphate buffer (100 mM, pH 7.4), L-aspartate (100 mM), and α-oxoglutarate (2 mM) and the mixture incubated for exactly 30 min at 37 ^ο^C Five hundred microliters (500 μL) of 2,4-dinitrphenylhydrazine (2 mM) was added to the reaction mixture and allowed to stand for exactly 20 min at 25 ^ο^C. Thereafter, 5.0 mL of NaOH (0.4 M) was added and left to stand for 5 min. Absorbance was then read at 546 nm against the reagent blank. AST activity was then obtained by interpolation.

### Assay of alanine amino transferase (ALT) activity

The principle previously described Reitman and Frankel [31] was followed in the assay of ALT using Randox kit. Five hundred microliters (500 μL) of reagent 1 containing, phosphate buffer (100 mM, pH 7.4), L-alanine (200 mM) and α-oxoglutarate (2.0 M) was added to 0.1 mL of serum and each of the organ homogenates in a test tube and the mixture was incubated at 37 ^ο^C for 30 min. Exactly 0.5 mL of reagent 2 containing, 2, 4-dinitrophenylhydrazine (2.0 mM) was added and the solution incubated again at 20 ^ο^C for 20 min. Finally, 5.0 mL of NaOH was added and the solution was allowed to stand for 5 min at room temperature and the absorbance measured at 546 nm. ALT activity was then obtained by interpolation.

### Assay of alkaline phosphatase (ALP) activity

Assay for ALP was based on the protocol described by Englehardt et al. [32]. Exactly 1.0 mL of the reagent (1.0 M diethanolamine buffer pH 9.8, 0.5 mM MgCl_2_; substrate: 10 mM p-nitrophenol phosphate) was added separately to 0.02 mL of serum and organs homogenates and mixed. The absorbance was taken at 405 nm for 3 min at 1 min interval. ALP activity was then obtained by interpolation.

### Estimation of Total cholesterol level

Total cholesterol level was determined based on established method of Trinder [33]. Ten microliter each of standard, serum and organs homogenates were pipetted into labeled test tubes. Thereafter, 1.0 mL of working reagent containing; Pipes buffer (80 mM pH 6.8), 4-aminoantipyrine (0.25 mM), phenol (6 mM), peroxidase (≥ 0.5 U/mL), cholesterol esterase ion (≥0.15 U/mL) and cholesterol oxidase (0.10 U/mL) was added into all the tubes. The test tubes were mixed thoroughly and incubated for 10 min at room temperature. Absorbance of the sample (Asample) was read at 500 nm against the reagent blank.

$$\mathrm{Cholesterol}\ \mathrm{concentration}\ \left(\mathrm{mg}/\mathrm{dL}\right)=\frac{\mathrm{Absorbance}\ \mathrm{of}\ \mathrm{sample}\kern1.25em }{\ \mathrm{Absorbance}\ \mathrm{of}\ \mathrm{standard}}\ \mathrm{X}\ \mathrm{Concentration}\ \mathrm{of}\ \mathrm{standard}$$

### Evaluation of concentration of triglyceride

Triglycerides level was measured as previously described by Tietz [34]. Ten microliters each of triglyceride standard, serum and organs homogenates were pipetted separately into labeled test tubes. Exactly 1.0 mL of the working reagents; R1a (buffer) containing Pipes buffer (40 mM, pH 7.6), 4-chloro-phenol (5.5 mM), magnesium-ion (17.5 mM); R1b (enzyme reagent containing 4-amino phenazone (0.5 mM), ATP (1.0 mM), lipase (≥150 U/mL), glycerol-kinase (≥0.4 U/ml), glycerol-3-phosphate oxidase (≥1.5 U/mL) and peroxidase (≥0.5 U/mL) was added into all the tubes. The reaction mixtures were mixed thoroughly and incubated for 10 min at room temperature. Absorbance was measured at 546 nm against the blank.

$$\mathrm{Triglyceride}\ \mathrm{concentration}\ \left(\mathrm{mg}/\mathrm{dL}\right)=\frac{\mathrm{Absorbance}\ \mathrm{of}\ \mathrm{sample}\kern1.25em }{\kern0.5em \mathrm{Absorbance}\ \mathrm{of}\ \mathrm{standard}}\ \mathrm{X}\ \mathrm{Concentration}\ \mathrm{of}\ \mathrm{standard}$$

### High density lipoprotein (HDL-c)-cholesterol assay

Estimation of HDL-cholesterol was done as described by Grove [35]. Reaction mixture containing 200 μL each of the serum and organs homogenates, 200 μL of the cholesterol standard, 500 μL of the diluted precipitant R1 (0.55 mM phosphotungstic acid, 25 mM magnesium chloride) were mixed together and allowed to stand for 10 min at room temperature. It was then centrifuged for 10 min at 4000 rpm to obtain a clear supernatant. The clear supernatant was separated off within 2 h and the cholesterol content was determined by the CHOD-PAP reaction method as follows:

One milliliter cholesterol reagent was added separately to 100 μL each of serum and organs homogenates and mixed together in a test tube. The standard test tube contained 100 μL of the cholesterol standard supernatant and 1 mL of cholesterol reagent. The reagent mixture was mixed thoroughly and incubated for 10 min at 25 ^ο^C. Absorbance of the sample (A _sample_) and standard (A_standard_) was then measured at 500 nm against the reagent blank within 1 h.

### Low density lipoprotein (LDL) - cholesterol determination

Concentration of low-density lipoprotein in the serum and organs homogenates was calculated as described as described by Friedwald et al. [36]:

$$\mathrm{LDL}\ \mathrm{cholesterol}=\mathrm{Total}\ \mathrm{cholesterol}-\frac{\mathrm{Triglycerides}}{5}-\mathrm{HDL}-\mathrm{cholesterol}$$

### Determination of catalase activity

Catalase activity was measured using the method previously described by Sinha [37]. Briefly, 200 μL each of serum and organs homogenates was mixed separately with 0.8 mL distilled H_2_O to obtain a five-fold dilution. The assay mixture contained 2 mL of solution (800 μmol) and 2.5 ml of phosphate buffer in a 10 mL flat bottom flask. Five hundred microliters of appropriate dilution of the enzyme was rapidly mixed with the reaction mixture by a gentle swirling motion at room temperature. Thereafter, 1.0 mL portion of the reaction mixture was withdrawn and blown into 1 mL dichromate/acetic acid reagent at 60 s intervals. Hydrogen peroxide content of the withdrawn sample was determined by the method described below.

$$\mathrm{Catalase}\kern0.17em \mathrm{activity}=\frac{{\mathrm{H}}_2{\mathrm{O}}_2\;\mathrm{Consumed}}{\mathrm{mg}\;\mathrm{protein}.}$$

$${\mathrm{H}}_2{\mathrm{O}}_2\ \mathrm{consumed}=800-\mathrm{Concentration}\ \mathrm{of}\ {\mathrm{H}}_2{\mathrm{O}}_2\ \mathrm{remaining}$$

Concentration of H_2_O_2_ remaining was extrapolated from the standard curve for catalase activity.

### Determination of superoxide dismutase (SOD) activity

Superoxide dismutase activity was determined following the method of Misra and Fridovich [38]. An aliquot of a ten-fold dilution each of serum and organs homogenates was added separately to 2.5 mL of 0.05 M carbonate buffer (pH 10.2) and allowed to equilibrate in a spectrophotometer. Reaction was initiated by the addition of 0.3 mL of freshly prepared 0.3 mM adrenaline to the mixture which was quickly mixed by inversion. The reference cuvette contained 2.5 mL buffer, 0.3 mL of substrate (adrenaline) and 0.2 mL of water. Absorbance at 480 nm of the resulting solution was monitored for 150 s at 30 s interval.

### Determination of reduced glutathione (GSH) level

Reduced glutathione was estimated according to the method of Beutler et al. [39] was followed in estimating the level of reduced glutathione (GSH). Exactly 0.2 mL each of serum and organs homogenates was added separately to 1.8 mL of distilled water followed by the addition of 3.0 mL of the precipitating solution and then shaken thoroughly. The mixture was then allowed to stand for 5 min and then filtered. One milliliter of filtrate was added of 4.0 mL of 0.1 M phosphate buffer pH 7.4. Finally, 0.5 mL of the Ellman’s reagent was added to the mixture. A blank was prepared with 4.0 mL of the 0.1 M phosphate buffer, 1.0 mL of diluted precipitating solution and 0.5 mL of the Ellman’s reagent. Absorbance of the resulting solution was then measured at 412 nm against reagent blank.

### Determination of Total protein (TP) in serum

Biuret method described by Weichselbaum [40] was employed in the determination of total protein. One milliliter of reagent R1 containing sodium hydroxide (100 mM), Na-K-tartrate (18 mM), potassium iodide (15 mM) and cupric sulphate (6 mM) was added separately to 0.02 mL each of serum and organs homogenates. The resulting mixture was incubated at 25 ^ο^C and absorbance measured at 546 nm against the reagent blank.

### Lipid peroxidation

Thiobarbituric acid reactive substances (TBARS) content in the serum and organs homogenates were measured as previously described by Okhawa et al. [41] using Randox kits. One hundred microliters each of serum and organs homogenates were mixed separately with 2.5 mL reaction buffer and boiled for 1 h. The resulting mixture was centrifuged at 3000 rpm for 10 min. Absorbance of the supernatant obtained for each tube was then measured at 532 nm. Malonidialdehyde (MDA) level in the supernatant was expressed as μmole MDA/mg protein using molar extinction coefficient of MDA-thiobarbituric chromophore (1.56 × 10^5^/M/cm).

### Statistical analysis

Data are expressed as mean ± SEM. Statistical evaluation was done using One-Way Analysis of Variance (ANOVA) followed by Duncan’s Multiple Range Test (DMRT) by using SPSS 11.09 for windows. The significance level was set at *p* < 0.05.

## Results

Generally, exposure of animals to CCl_4_ and rifampicin caused 60 and 73% increase respectively in hepatic cholesterol relative to the negative control (Table 1). The trend was similar for LDL and triglyceride (Table 1). Hepatic HDL level was depleted by 33 and 25% following exposure to CCl_4_ and rifampicin respectively. A similar trend was observed in the kidney and serum. Treatment with *M. pruriens* extract brought about 107 and 98% restoration in lipid profile in the liver of animals challenged with CCl_4_ and rifampicin respectively relative to negative control (animals that were not exposed at all) and 108% for animals treated with the standard drug. The restoration was dose-dependent (Table 1). Aspartate transaminase (AST) activities in the liver were increased by 52 and 78% following exposure to CCl_4_ and rifampicin respectively. Alkaline phosphatase (ALP) and alanine transaminase (ALT) activity also increased by similar percentage as AST when animals were exposed to CCl_4_ and rifampicin irrespective of the tissues (kidney or serum) involved (Table 2). However, oral treatment with *M. pruriens* extract at 100 mg/kg bw restored AST level in the liver by 102 and 92% for animals induced with CCl_4_ and rifampicin respectively relative to negative control. Silymarin at 100 mg/kg bw ameliorated hepatic damage by 95% which is lower than the effect caused by *M. pruriens* leaf extract at the same dose (Table 2). Percentage restoration was similar regardless of the tissue involved and toxicant employed for induction of oxidative assault.

**Table 1:** Animal Treatment

Groups	Treatment
I: Negative Control	Distilled water only
II: Positive Control	3 ml CCl_4_ single administration
III	3 ml CCl_4_ + 50 mg/kg b.w *M. pruriens*
IV	3 ml CCl_4_ + 100 mg/kg b.w. *M. pruriens*
V	3 ml CCl_4_ + 100 mg/kg b.w. *Silymarin*
VI	250 mg/kg b.w. rifampicin alone
VII	250 mg/kg b.w. rifampicin + 50 mg/kg *M. pruriens*
VIII	250 mg/kg b.w. rifampicin + 100 mg/kg *M. pruriens*

**Table 2:** Effects of *M. pruriens* extract on lipid profile in the liver and kidney of rats after CCl_4_ and rifampicin induced toxicity

Parameters	Tissues	Negative Control	Positive Control (CCl_4_ only)	CCl_4_ + 50 mg/kg b.w MP	CCl_4_ + 100 mg/kg b.w MP	CCl_4_ + 100 mg/kg b.w Silymarin	Negative Control II (Rifampicin only)	Rifampicin + 50 mg/kg b.w MP	Rifampicin + 100 mg/kg b.w MP
T. Chol (mg/dl)	Liver	56.08 ± 2.09^a^	89.12 ± 1.73	64.22 ± 1.23^a^	53.25 ± 1.06^a^	52.33 ± 1.34^a^	97.23 ± 1.42	66.40 ± 2.15^a^	58.29 ± 1.76^a^
	Kidney	30.07 ± 2.18^a^	41.86 ± 2.09	33.22 ± 2.03^a^	27.63 ± 1.97^a^	38.63 ± 2.23^a^	53.24 ± 1.03	46.32 ± 1.23^a^	34.04 ± 1.16^a^
	Serum	52.16 ± 2.19^a^	72.44 ± 1.86	61.32 ± 1.36^a^	56.82 ± 1.45^a^	58.27 ± 1.56^a^	87.46 ± 1.38	64.03 ± 1.02^a^	59.34 ± 2.16^a^
Trig. (mg/dl)	Liver	41.33 ± 1.07^a^	70.25 ± 1.24	51.27 ± 1.39^a^	43.76 ± 1.22^a^	39.43 ± 1.13^a^	86.50 ± 0.42	71.49 ± 0.63^a^	60.08 ± 1.23^a^
	Kidney	11.82 ± 0.34^a^	19.47 ± 0.42	14.06 ± 0.77^a^	12.93 ± 0.64^a^	13.43 ± 0.29^a^	26.48 ± 0.33	20.13 ± 0.29^a^	16.25 ± 0.20^a^
	Serum	37.51 ± 1.39^a^	61.41 ± 1.28	50.14 ± 1.07^a^	42.66 ± 1.33^a^	40.14 ± 1.72^a^	70.39 ± 1.45	53.86 ± 2.09^a^	46.25 ± 1.77^a^
HDL (mg/dl)	Liver	24.72 ± 0.44^a^	16.07 ± 0.53	20.15 ± 0.68^a^	22.43 ± 0.53^a^	26.31 ± 0.59^a^	18.26 ± 0.24	21.72 ± 0.66^a^	23.18 ± 0.52^a^
	Kidney	8.26 ± 0.17^a^	5.31 ± 0.87	8.12 ± 0.56^a^	8.51 ± 0.53^a^	8.42 ± 0.68^a^	6.08 ± 0.63	6.83 ± 0.53^a^	7.27 ± 0.76^a^
	Serum	9.42 ± 0.31^a^	6.37 ± 0.43	7.00 ± 0.81^a^	7.82 ± 0.70^a^	8.79 ± 0.29^a^	5.10 ± 0.07	8.15 ± 0.05^a^	8.86 ± 0.02^a^
LDL (mg/dl)	Liver	33.76 ± 1.27^a^	41.27 ± 1.53	36.09 ± 1.31^a^	32.47 ± 0.78^a^	33.04 ± 0.62^a^	57.81 ± 0.11	48.06 ± 0.37^a^	39.16 ± 0.25^a^
	Kidney	13.26 ± 0.73^a^	21.60 ± 0.82	16.01 ± 0.69^a^	14.07 ± 0.43^a^	12.13 ± 0.48^a^	31.27 ± 0.57	24.12 ± 0.54^a^	19.33 ± 0.66^a^
	Serum	22.36 ± 0.32^a^	34.26 ± 0.53	26.42 ± 0.34^a^	22.14 ± 0.13^a^	24.67 ± 0.72^a^	39.53 ± 0.68	30.06 ± 0.51^a^	23.72 ± 0.44^a^

Data shows mean ± SEM values of four independent experiments performed in triplicate ‘a’ represents significant difference (*p* < 0.05) from the control, (*n* = 5)

Activity of hepatic catalase was inhibited 35 and 65% in animals administered with CCl_4_ and rifampicin respectively. Treatment with the extract at 100 mg/kg bw reversed the inhibition by 82 and 88% for CCl_4_ and rifampicin respectively, while silymarin relieved the inhibition by 85% which is comparatively lower to effect caused by the extract at the same dose. A similar trend was observed for superoxide dismutase regardless of the tissue involved and toxicant employed for oxidative assault (Table 3). Endogenous antioxidant tripeptide, reduced glutathione (GSH) was depleted by 33, 22 and 41% in the liver, kidney and serum respectively while 49, 10 and 22% depletion in GSH was observed when animals were exposed to CCl_4_ and rifampicin respectively (Table 3). Treatment with *M. pruriens* extract caused a dose-dependent restoration of hepatic GSH level (73% at 50 mg/kg bw; 88% at 100 mg/kg bw) and (55% at 50 mg/kg bw; 73% at 100 mg/kg bw) for CCl_4_ and rifampicin respectively relative to negative control. Noteworthy is the fact that treatment with 100 mg/kg bw silymarin caused 94% restoration of hepatic GSH relative to negative control (Table 3). Table 4 shows that CCl_4_ and rifampicin caused a 60 and 47% increase respectively in renal urea relative to normal rats. After treatment with 100 mg/kg silymarin, renal urea was reduced to 12%, while treatment with 50 and 100 mg/kg bw *M. pruriens* caused urea level to be brought from 60 to 28 and 2% respectively for animals exposed to CCl_4_. Similarly, urea level was reduced from 47% to 29 and 14% respectively relative to the negative control. Notably, the extract produced a stronger effect than silymarin, the standard drug (Table 4). The same trend was observed for uric acid and creatine kinase in the kidney and serum of experimental animals (Table 4). Lipid peroxidation was increased by 83, 108 and 142% in the liver, kidney and serum respectively following exposure to 3 ml/kg bw CCl_4_ while 152, 105 and 63% in the liver, kidney and serum respectively of animals exposed to 250 mg/kg bw rifampicin. Treatment with silymarin produced a 100% inhibition of hepatic lipid peroxidation relative to negative control animals. Treatment with 50 and 100 mg/kg bw *M. pruriens* resulted in 48 and 91% inhibition in hepatic lipid peroxidation respectively for animals exposed to CCl_4_ toxicity. On the other hand, 43 and 91% inhibition were observed respectively when animals exposed to rifampicin toxicity were treated with 50 and 100 mg/kg bw *M. pruriens* (Table 5). The trend was similar for kidney and serum lipid peroxidation.

**Table 3:** Effects of *M. pruriens* extract on selected biomarkers (AST, ALT, ALP and T. BIL.) in the liver and kidney of rats after CCl_4_ and rifampicin induced toxicity

Parameters	Tissues	Negative Control I	Positive Control (CCl_4_ only)	CCl_4_ + 50 mg/kg b.w MP	CCl_4_ + 100 mg/kg b.w MP	CCl_4_ + 100 mg/kg b.w Silymarin	Negative Control II (Rifampicin only)	Rifampicin + 50 mg/kg b.w MP	Rifampicin + 100 mg/kg b.w MP
AST (IU/l)	Liver	54.33 ± 2.31^a^	82.31 ± 2.47	62.13 ± 0.14^a^	52.93 ± 0.10^a^	57.23 ± 1.53^a^	96.14 ± 2.41	71.52 ± 2.15^a^	58.77 ± 1.87^a^
	Kidney	21.60 ± 2.07^a^	37.81 ± 1.02	30.60 ± 1.12^a^	26.34 ± 1.08^a^	25.06 ± 1.20^a^	56.82 ± 1.73	38.62 ± 1.63^a^	28.26 ± 1.29^a^
	Serum	71.26 ± 1.71^a^	115.63 ± 1.67	93.74 ± 1.88^a^	78.14 ± 0.98^a^	75.22 ± 1.56^a^	127.13 ± 2.60	112.31 ± 3.11^a^	92.14 ± 2.34^a^
ALT (IU/l)	Liver	40.71 ± 1.32^a^	64.21 ± 1.43	55.81 ± 1.16^a^	50.04 ± 2.01^a^	51.33 ± 1.1^a^	77.23 ± 1.42	62.49 ± 1.98^a^	42.03 ± 1.58^a^
	Kidney	17.27 ± 0.25^a^	30.44 ± 0.97	24.16 ± 0.84^a^	20.03 ± 0.29^a^	23.63 ± 1.23^a^	40.29 ± 1.77	36.29 ± 1.23^a^	23.60 ± 2.01^a^
	Serum	56.17 ± 1.98^a^	92.76 ± 1.23	72.10 ± 2.13^a^	64.40 ± 2.22^a^	59.33 ± 1.34^a^	88.45 ± 2.43	62.75 ± 2.00^a^	59.22 ± 1.96^a^
ALP (IU/l)	Liver	47.25 ± 1.92^a^	58.78 ± 1.63	51.06 ± 1.35	42.53 ± 1.07^a^	46.88 ± 0.49^a^	73.84 ± 0.81	64.67 ± 0.92^a^	49.22 ± 0.67^a^
	Kidney	31.21 ± 1.25^a^	48.17 ± 0.84	33.09 ± 0.92^a^	26.04 ± 0.88^a^	29.32 ± 0.98^a^	53.66 ± 1.98	44.36 ± 1.72^a^	34.83 ± 1.45^a^
	Serum	62.13 ± 3.12 ^a^	102.43 ± 4.20	82.42 ± 2.00^a^	71.38 ± 1.86^a^	67.11 ± 2.09^a^	108.52 ± 1.32	81.57 ± 2.07^a^	68.34 ± 1.82^a^
T. Bil	Liver	31.18 ± 4.04^a^	44.37 ± 3.54	37.25 ± 2.10^a^	30.34 ± 2.08^a^	31.72 ± 0.82^a^	56.18 ± 0.54	42.33 ± 0.61^a^	32.40 ± 0.50^a^
	Serum	42.25 ± 1.43^a^	72.18 ± 1.54	58.93 ± 1.43^a^	48.52 ± 1.37^a^	45.37 ± 1.84^a^	69.30 ± 1.02	53.26 ± 2.01^a^	43.17 ± 1.33^a^

Data shows mean ± SEM values of four independent experiments performed in triplicate ‘a’ represents significant difference (*p* < 0.05) from the control, (*n* = 5)

**Table 4:** Effects of *M. pruriens* extract on selected antioxidant enzymes (superoxide dismutase and Catalase) and reduced glutathione (GSH) in the liver and kidney of rats after CCl_4_ and rifampicin induced toxicity

Parameters	Tissues	Negative Control	Positive Control (CCl_4_ only)	CCl_4_ + 50 mg/kg b.w MP	CCl_4_ + 100 mg/kg b.w MP	CCl_4_ + 100 mg/kg b.w Silymarin	Negative Control II (Rifampicin only)	Rifampicin + 50 mg/kg b.w MP	Rifampicin + 100 mg/kg b.w MP
SOD (μmol/min/mg protein)	Liver	6.14 ± 0.31^a^	4.53 ± 0.22	4.72 ± 0.61^a^	4.93 ± 0.31^a^	6.20 ± 1.23^a^	2.36 ± 0.19	2.75 ± 0.12^a^	3.89 ± 0.15^a^
	Kidney	3.48 ± 1.03^a^	2.46 ± 0.75	2.61 ± 0.54^a^	2.90 ± 0.44^a^	3.15 ± 0.53^a^	1.93 ± 0.19	2.76 ± 0.41^a^	3.08 ± 0.26^a^
	Serum	4.29 ± 0.19^a^	3.17 ± 0.10	3.28 ± 0.18^a^	4.00 ± 0.33^a^	4.34 ± 0.21^a^	2.42 ± 0.07	2.89 ± 0.13^a^	3.76 ± 0.21^a^
Catalase (μmol/min/mg protein)	Liver	4.70 ± 0.56^a^	2.89 ± 0.21	3.15 ± 0.11^a^	4.15 ± 0.30^a^	3.97 ± 1.20^a^	1.63 ± 0.16	2.60 ± 0.41^a^	3.84 ± 0.23^a^
	Kidney	2.77 ± 0.15^a^	2.02 ± 0.18	2.32 ± 0.20^a^	2.47 ± 0.11^a^	2.58 ± 0.20^a^	1.88 ± 0.26	2.16 ± 0.12^a^	2.53 ± 0.10^a^
	Serum	3.06 ± 0.11^a^	2.38 ± 0.10	2.66 ± 0.14^a^	2.91 ± 0.10^a^	2.93 ± 0.20^a^	1.76 ± 0.14	2.53 ± 0.20^a^	3.01 ± 0.16^a^
GSH ((μmol/mg protein)	Liver	5.77 ± 0.06^a^	3.87 ± 0.01	4.20 ± 0.12^a^	5.13 ± 0.10^a^	5.43 ± 0.08^a^	2.93 ± 0.08	3.17 ± 0.02^a^	4.22 ± 0.06^a^
	Kidney	3.23 ± 0.07^a^	2.53 ± 0.05	2.88 ± 0.08^a^	3.07 ± 0.09^a^	3.19 ± 0.12^a^	3.04 ± 0.02	3.15 ± 0.08^a^	3.19 ± 0.10^a^
	Serum	2.66 ± 0.10^a^	1.57 ± 0.22	2.07 ± 0.64^a^	2.10 ± 0.52^a^	2.49 ± 0.98^a^	2.08 ± 0.02	2.30 ± 0.04^a^	2.52 ± 0.01^a^

Data shows mean ± SEM values of four independent experiments performed in triplicate ‘a’ represents significant difference (*p* < 0.05) from the control, (*n* = 5)

**Table 5:** Effects of *M. pruriens* extract on selected biomarkers (urea, uric acid and creatine kinase in the liver and kidney of rats after exposure to CCl_4_ and rifampicin toxicity

Parameters	Tissues	Negative Control	Positive Control (CCl_4_ only)	CCl_4_ + 50 mg/kg b.w MP	CCl_4_ + 100 mg/kg b.w MP	CCl_4_ + 100 mg/kg b.w Silymarin	Negative Control II (Rifampicin only)	Rifampicin + 50 mg/kg b.w MP	Rifampicin + 100 mg/kg b.w MP
Urea (mg/dl)	Kidney	47.82 ± 0.93^a^	76.32 ± 0.88	61.37 ± 1.34^a^	48.93 ± 1.17^a^	53.76 ± 0.78^a^	70.06 ± 0.83	61.82 ± 0.51^a^	54.70 ± 1.20^a^
	Serum	38.77 ± 0.83^a^	77.64 ± 1.36	59.26 ± 1.06^a^	51.26 ± 0.96^a^	45.26 ± 0.76^a^	64.39 ± 0.62	53.14 ± 0.76^a^	41.20 ± 0.81^a^
Uric acid (mg/dl)	Kidney	29.54 ± 1.52^a^	42.65 ± 0.82	33.16 ± 0.55^a^	32.02 ± 0.87^a^	32.89 ± 0.92^a^	54.23 ± 0.78	43.06 ± 1.08^a^	31.37 ± 1.13^a^
	Serum	19.17 ± 0.24^a^	33.51 ± 0.52	28.47 ± 0.65^a^	24.81 ± 0.59^a^	21.17 ± 0.68^a^	35.43 ± 0.52	28.76 ± 0.48^a^	24.08 ± 0.53^a^
Creatine kinase (IU/L)	Kidney	27.61 ± 1.07^a^	38.44 ± 0.87	30.22 ± 0.19^a^	28.31 ± 0.14^a^	25.73 ± 0.85^a^	49.17 ± 0.94	40.80 ± 0.64^a^	33.51 ± 0.50^a^
	Serum	20.33 ± 0.30^a^	31.70 ± 0.29	26.31 ± 1.00^a^	22.40 ± 0.81^a^	23.66 ± 0.22^a^	44.58 ± 0.44	31.49 ± 0.42^a^	28.60 ± 0.58^a^

Data shows mean ± SEM values of four independent experiments performed in triplicate ‘a’ represents significant difference (*p* < 0.05) from the control, (*n* = 5)

## Discussion

The onset, progression and complications of several pathological conditions have been linked to oxidative stress which can been modelled experimentally for robust research investigation [1, 2, 42–44]. In the present study (Table 1), exposure to CCl_4_ resulted in a significant derangement in lipid profile of experimental animals. Total cholesterol, triglycerides and LDL were significantly elevated while HDL level was markedly depleted compared to negative control animals. This result is in agreement with Agbafor and Nwachukwu, [45]. The molecular event of CCl_4_-induced hepatic damage involves the activation of several transcription factors such as NF-kB, activator protein 1 (AP-1) and early growth response 1 (EGR-1). When activated, NF-kB triggers an upregulation of the inflammatory cascade thereby releasing specific proinflammatory cytokines responsible for hepatic inflammation [24, 46]. The observed derangement in lipid profile can be attributed to oxidative stress. Treatment with *M. pruriens* extract, restored the lipid profile of intoxicated experimental rats in a dose-dependent fashion comparable to silymarin, the standard drug. This observation is in agreement with Enechi and Ozogwu, [47] and suggests the potential of *M. pruriens* leaf extract in the management of diseases. Divya et al. [48], Yadav et al. [49] had earlier identified the phytochemical constituents of *Mucuna pruriens* leaves as anthraquinones, flavonoids and cardiac glycosides using preliminary phytochemical; screening. More recently, detailed phytochemical constituents of ethanolic extract of *Mucuna pruriens* leaf was reported by Ezim et al. [50] using GC-MS analysis. Previous reports suggested that the antihyperlipidemic potentials of plant extracts is due to the presence of these phytochemicals in their extracts [51]. Flavonoids have been reported to lower the levels of triglycerides and cholesterol in the blood thereby minimizing the risk of cardiovascular disorders such as atherosclerosis [52]. Saponins has affinity for cholesterol and bile salts. Hence, it binds them, thereby lowering their blood levels by inhibiting their reabsorption in the intestinal tract [53]. Cardiac glycosides act by altering the availability of intracellular Ca^2+^ for myocardial contraction [54]. The anti-hypercholesterolemic effect of the extract may be linked to suggested mechanisms such as the regulation of cholesterol biosynthesis; inhibition of LDL oxidation; inhibition of intestinal cholesterol absorption by complex formation.

Anti-inflammatory potentials of flavonoids and other polyphenols have been extensively studied [26]. Specifically, flavonoids act by down regulating inflammatory transcriptional factors via the activation of transcription factor-3, activator protein-1 and CREB binding proteins. Therefore, the hepatoprotective effect of *M. pruriens* leaf extract can be attributed to flavonoid-induced NF-kB inhibition, thereby restoring liver histoarchitecture and function [26]. Exposure to rifampicin caused a significant alteration in renal lipid profile of experimental animals (Table 2). Rifampicin toxicity in the kidney and liver involves lipid peroxidation as well as inhibition of key detoxification enzymes [55]. In the liver, rifampicin is bioactivated to deacetyl rifampicin, which binds to critical macromolecules causing liver and kidney injury [55–57]. Treatment with *M. pruriens* extract reversed the toxicity imposed on renal lipid profile in a dose dependent manner (Table 2). This observation is in agreement with the earlier report of Mohammed et al. [58]. This effect can be attributed to the presence of a significant level of flavonoids which scavenged the free radicals initially generated when experimental animals were exposed to the toxicants. Noteworthy is the fact that, extract of *M. pruriens* exhibited comparable potency to silymarin at the same concentration. This observation is attributable to the cocktail of antioxidant phytochemicals present in the extract [58]

Specific enzymes such as ALT, AST and ALP have been used as bio-indicators of liver functionality [58]. During organs damage, cellular enzymes such as AST, ALT, and ALP leak into the blood resulting in an elevation of their concentrations. In the present study, marked elevation in serum levels of ALP, AST and especially ALT suggested liver injury in the exposed rats without treatment (Table 3). However, treatment with graded doses of *M. pruriens* extract resulted in a dose-dependent restoration of ALT, AST and ALP levels in the blood and other tissues involved (Table 3). This observation is in agreement with Mohammed et al. [58] and Sylvester et al. [59]. The observed restoration is traceable to the presence of flavonoids, anthraquinones and other phytochemicals with established antioxidant potentials (Yadav et al., 2017) [49].

Antioxidant enzymes act as molecular shield for critical macromolecules, protecting them from free radicals’ attack. Hence, they are often used as indicators to monitor the health status of experimental animals. In the present study, significant decrease in superoxide dismutase and catalase was observed in animals challenged with CCl_4_ and rifampicin (Table 4). This is due to the fact that hepatotoxic and nephrotoxic metabolites of CCl_4_ and rifampicin metabolism respectively bind to critical functional groups of antioxidant enzymes leading to their inhibition [60]. Treatment of exposed animals with *M. pruriens* extract restored these parameters in the serum and selected organs in a manner comparable to silymarin(Table 4). This restoration is due to the presence of flavonoids present in the extract as earlier reported by Mohammed et al. [58]. The restorative mechanism is suggested to involve the polyphenolic components of *M. pruriens* extract preventing the toxic metabolites of rifampicin and CCl_4_ from binding to critical groups on antioxidant enzymes.

Serum concentration of bilirubin can provide useful clinical information about the health status of patients with hepatic disease. It has been suggested that elevated serum bilirubin is an evidence of protracted liver disease [61–64]. Exposure of animals to CCl_4_ and rifampicin respectively caused a significant increase in serum bilirubin, suggesting a potential hepatocellular injury. However, treatment with *M. pruriens* extract resulted in a dose-dependent restoration of bilirubin to a level comparable to animals that were not exposed to the toxicants. This observation is in agreement with Enechi and Ozogwu, [47]. The restorative effect can be attributed to the cocktail of antioxidant phytochemicals present in the extract.

Urea, a product of amino acid catabolism is a major bioindicator of the integrity and functionality of the kidney. It has been used as a diagnostic tool to monitor the efficiency of dialysis procedure in patients with chronic kidney disease [65]. CCl_4_ and rifampicin caused a significant increase in serum urea level of exposed animals relative to normal control (Table 5). This suggests a free radical-induced damage in the glomerulus and eventual dysfunction in ultrafiltration leading to urea retention. *M. pruriens* treatment restored the serum urea level in a dose-dependent manner comparable to normal animals and those treated with silymarin. This observation is in agreement with Enechi and Ozogwu [47]. This is an indication that *M. pruriens* offers a promising potential in the management of kidney diseases.

Measurement of uric acid as a routine parameter is traceable to its role in the formation of gout, kidney diseases and type II diabetes [66]. Exposure to CCl_4_ and rifampicin caused a marked elevation in serum uric acid relative to animals that were not exposed to toxicants. This is attributed to free radical-induced alteration in purine metabolism. Treatment with *M. pruriens* extract restored the uric acid in the serum and organs homogenates to a level similar to animals treated with silymarin (Table 5). The restorative potential of *M. pruriens* was due to possible inhibition of an upregulation of purine catabolism, an activity attributable to the polyphenolic components of the extract.

Elevation in serum creatine kinase, a muscle enzyme released during myocardial necrosis is an indication of cardiac injury. In the present study, serum creatine kinase was significantly increased in animals exposed to toxicants (Table 5). This observation suggests that the toxicants exhibit multi-organ toxicity. Worthy of note is the fact that extract of *M. pruriens* leaf ameliorated the process restoring creatine kinase activity to a level comparable with animals treated with silymarin. This effect is due to a cocktail of antioxidant phytochemicals present in the extract.

The devastating role of lipid peroxidation on human health has been suggested [60]. Exposure to CCl_4_ and rifampicin resulted in a significant increase in malonidialdehyde (MDA) content in the serum and tissue homogenates of experimental animals (Table 6). This observation was due to metabolism of CCl_4_ to hepatotoxic intermediates such as trichloromethyl and trichloroperoxyl radicals which are the major culprits in lipid peroxidation [60]. Treatment with *M. pruriens* extract caused a dose-dependent inhibition of lipid peroxidation with potency comparable to silymarin. This effect is due to the additive or synergistic interactions of the various antioxidant phytochemicals in the leaf extract of *M. pruriens*. It therefore suggests that leaf extract of *M. pruriens* is a potential therapeutic alternative in the management of kidney and liver related diseases.

**Table 6:** Effects of *M. pruriens* extract on lipid peroxidation (MDA) level and total protein in the liver and kidney of rats after CCl_4_ and rifampicin induced toxicity

Parameters	Tissues	Negative Control	Positive Control (CCl_4_ only)	CCl_4_ + 50 mg/kg b.w MP	CCl_4_ + 100 mg/kg b.w MP	CCl_4_ + 100 mg/kg b.w Silymarin	Negative Control II (Rifampicin only)	Rifampicin + 50 mg/kg b.w MP	Rifampicin + 100 mg/kg b.w MP
Malonidialdehyde (MDA) (mM/g tissue)	Liver	0.23 ± 0.01^a^	0.42 ± 0.01	0.35 ± 0.01^a^	0.25 ± 0.01^a^	0.20 ± 0.01^a^	0.58 ± 0.01	0.36 ± 0.02^a^	0.25 ± 0.02^a^
	Kidney	0.89 ± 0.01^a^	1.76 ± 0.03	1.23 ± 0.01^a^	1.09 ± 0.02^a^	1.02 ± 0.01^a^	0.94 ± 0.01	0.52 ± 0.01^a^	0.47 ± 0.03^a^
	Serum	0.64 ± 0.02^a^	1.55 ± 0.01^a^	0.70 ± 0.02^a^	0.60 ± 0.07^a^	0.59 ± 0.04^a^	1.04 ± 0.09	0.63 ± 0.01^a^	0.58 ± 0.01^a^
Total Protein (mg/g tissue)	Liver	2.64 ± 0.16^a^	1.47 ± 0.19	2.07 ± 0.42^a^	2.15 ± 0.17^a^	2.33 ± 0.78^a^	1.04 ± 0.09	1.63 ± 0.02^a^	2.53 ± 0.01^a^
	Kidney	1.73 ± 0.020^a^	0.76 ± 0.06	0.89 ± 0.01^a^	1.24 ± 0.01^a^	1.44 ± 0.07^a^	1.06 ± 0.01	1.20 ± 0.01^a^	1.46 ± 0.02^a^
	Serum	2.08 ± 0.08^a^	1.26 ± 0.06	1.81 ± 0.01^a^	1.74 ± 0.01^a^	2.17 ± 0.08^a^	1.02 ± 0.18	1.15 ± 0.13^a^	1.88 ± 0.20^a^

Data shows mean ± SEM values of four independent experiments performed in triplicate ‘a’ represents significant difference (*p* < 0.05) from the control, (*n* = 5). Group I- Positive control; Group II- negative control (CCl_4_); Group III – Animals treated with M. pruriens extract at 50 mg/kg bw after exposure to CCl_4_; Group IV – Animals treated with M. pruriens (100 mg/kg bw); Group V- Animals treated with silymarin (100 mg/kg bw) after exposure to CCl_4_. Group VI- Negative control (rifampicin). Group VII- Animals treated with M. pruriens (50 mg/kg bw) extract after exposure to rifampicin; Group VIII- Animals treated with M. pruriens (100 mg/kg bw) after exposure to rifampicin

**Fig. 1:**
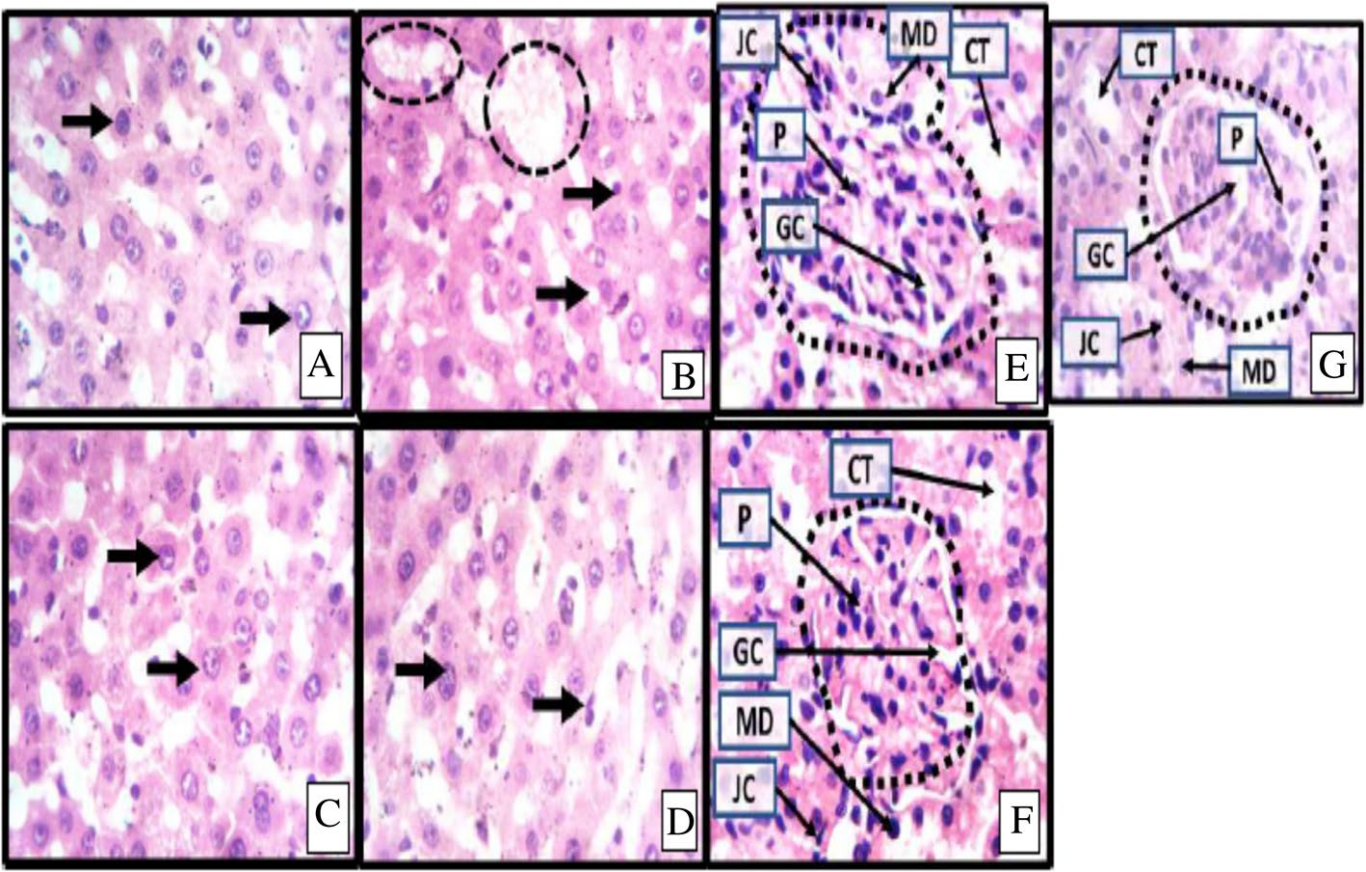
Representative photomicrograph of the liver (**A-D**) and kidney (**E-G**) slices of experimental animals. **A-D**, showing a high-power magnification (× 400 mag) of the inherent hepatocytes (black arrow head). Photomicrographs show the histomorphological manifestation of the hepatocytes, density of hepatocytes, distribution of hepatocytes, staining intensity of hepatocytes, size of central veins, content of central veins and expression of large vacuolations (dotted black circles). Large vacoulations with pick colouration are fatty livers with bile plaques which suggests cholestasis. Similarly, **E-G** showing a high-power magnification (x400mag) of renal corpuscle (black outline) which houses the glomerulus within the urinary space that is supplied by the afferent arteriole and drained by the efferent arteriole. The histomorphology presents with the convoluted tubule (CT), glomerular capillaries (GC) and inherent cells which include the intraglomerular podocytes (P) as well as the juxtaglomerular cells and macula densa cells in the vascular poles of the renal corpuscles. The urinary pole continues out as the proximal convoluted tubules. A- liver slice of animals fed with animal feed and distilled water only; B- liver slice of animals administered with 3 ml/kg CCl_4_, without treatment; C- liver slice of animals induced with 3 ml/kg CCl_4_ and treated with 100 mg/kg M. pruriens; D - liver slice of animals administered with 3 ml/kg CCl_4_ and treated with 200 mg/kg Silymarin; E - kidney slice of animals administered with distilled water only; F - kidney slice of animals administered with 250 mg/kg rifampicin only without treatment; G- kidney slice of animals administered with 250 mg/kg rifampicin and treated with 100 mg/kg bw of M. pruriens

Histopathological results (Fig. 1A-G), indicated a distortion in the liver parenchymal histoarchitecture which manifested as severe hepatocellular necrotic vacuolation, cellular inflammation and congestion of central vein in animals exposed to CCl_4_. This distortion can be attributed to free radical induced oxidative damage. These findings are in agreement with previous report [67]. Treatment with *M. pruriens* caused a restoration of the hepatic histoarchitecture in a manner similar to silymarin-treated animals. Hence, extract of *M. pruriens* leaf protects against membrane permeability and remedied drug-induced histopathological distortions. This observation is consistent with earlier reports [68–70].

## Conclusion

*M. pruriens* leaf extract inhibited lipid peroxidation, restored deranged lipid profile, improved liver and kidney biomarkers, restored antioxidant enzymes activity and ameliorated distorted liver and kidney histoarchitecture. Biochemical and histopathological parameters showed that *M. pruriens* leaf extract is as potent as silymarin at the same dose. Hence, it can favorably compete with conventional drugs available for the treatment of liver and kidney diseases. Further studies should therefore involve detailed phytochemical constituents of the extract as well as identifying the active principle in the extract and its mechanism of action.

## Data Availability

Data and materials related to the present work will be made available on request by the corresponding author
